# Dietary magnesium intake and dementia risk in community-dwelling people aged 40–74 years: an 8-year cohort study

**DOI:** 10.1017/jns.2025.10075

**Published:** 2026-01-22

**Authors:** Irina Bulycheva, Yumi Watanabe, Kaori Kitamura, Keiko Kabasawa, Toshiko Saito, Akemi Takahashi, Ryosaku Kobayashi, Rieko Oshiki, Ribeka Takachi, Shoichiro Tsugane, Osamu Yamazaki, Kei Watanabe, Kazutoshi Nakamura

**Affiliations:** 1 Division of Preventive Medicine, Niigata University Graduate School of Medical and Dental Sciences, Niigata, Japan; 2 Department of Health Promotion Medicine, Niigata University Graduate School of Medical and Dental Sciences, Niigata, Japan; 3 Department of Rehabilitation, Niigata University of Rehabilitation, Niigata, Japan; 4 Department of Food Science and Nutrition, Nara Women’s University Graduate School of Humanities and Sciences, Nara, Japan; 5 International University of Health and Welfare Graduate School of Public Health, Tokyo, Japan; 6 Niigata Prefectural Government, Niigata, Japan; 7 Division of Orthopedic Surgery, Niigata University Graduate School of Medical and Dental Sciences, Niigata, Japan

**Keywords:** Alzheimer’s disease, Cohort study, Dementia, Diet, Magnesium, AD, Alzheimer’s disease, BMI, body mass index, FFQ, food frequency questionnaire, HR, hazard ratio, IL, interleukin, LTCI, long-term care insurance, Mg, magnesium, MET, metabolic equivalent, NMDA, N-methyl D-aspartate, PA, physical activity, TNF-α, tumor necrosis factor-α

## Abstract

Dietary magnesium (Mg) is a potentially modifiable factor in preventing dementia, but current evidence supporting this remains insufficient and inconclusive. This study aimed to determine whether dietary Mg is associated with the risk of dementia among middle-aged and older people. Participants of this 8-year cohort study were 13,032 community-dwelling individuals aged 40–74 years. Dietary data were collected using a validated food frequency questionnaire in 2011–2013. Mg intake was adjusted for energy intake using the residual method. The outcome was newly diagnosed dementia determined using Japan’s long-term care insurance database. Covariates included demographic characteristics, body size, lifestyles, and disease histories. Cox proportional hazard models were used to determine adjusted hazard ratios (HRs). The mean age of participants was 59.0 years. Dementia occurred in 148 males and 138 females. Lower quartiles of energy-adjusted Mg intake were associated with a higher risk of dementia (*P* for trend = 0.0410) in males, with the lowest quartile (Q1) having an elevated risk of dementia (HR = 1.73, 95% CI:1.07–2.83) compared to the highest quartile (Q4, reference); however, this association was not found in females. In a subgroup analysis by disease history in males, the HR of Q1 was attenuated in both subgroups; HR was 1.52 (95% CI:0.74–3.11) in those with a disease history and 1.40 (95% CI:0.73–2.69) in those without. In conclusion, low dietary Mg intake is associated with increased dementia risk in middle-aged and older Japanese males. However, this association may be partly attributable to underlying disease history.

## Introduction

Dementia is one of the leading causes of cognitive and functional decline among older adults worldwide and is of growing global health importance as population ageing continues and effective cures remain elusive.^(1)^ Globally, an estimated 55 million or more people were affected by dementia in 2021.^(2)^ According to the Global Burden of Disease Study 2019, the number of people with dementia will increase to 83.2 million in 2030, reaching 152.8 million by 2050.^(3)^ In Japan, the projected number of people living with dementia is estimated to increase from 4.1 million in 2019 to 5.2 million in 2050.^(3)^ Dementia impairs the daily activities and quality of life of patients and imposes a serious burden in terms of not only patient and caregiver well-being but also economic, medical, social care, and informal care costs. Indeed, the global costs of dementia were estimated to amount to US$ 1.3 trillion in 2019, with a projected increase to US$ 1.7 trillion by 2030; corrected projections including care costs will reach US$ 2.8 trillion in 2030.^(2)^ The prevention of dementia is thus one of the most urgent public health issues.

Nutrients are promising modifiable factors that can contribute to dementia prevention. Magnesium (Mg), an essential mineral, necessitates intake through diet or supplements and plays a critical role in nerve transmission and neuromuscular coordination.^(4)^ Mg deficiency leads to increased production of free radicals and oxidative tissue damage, along with reduced antioxidant enzyme activity.^(5)^ Higher brain Mg levels are reportedly beneficial for cognitive function, possibly through the direct regulation of neuronal Mg on N-methyl-D-aspartate (NMDA) receptors.^(6)^ An imbalance in brain Mg levels can disrupt the regulation of NMDA receptors, leading to increased excitotoxicity, neuronal damage, and subsequent synaptic loss, key factors in the pathogenesis of dementia, particularly Alzheimer’s disease (AD).^(5,7)^ Thus, insufficient Mg intake may increase the risk of dementia.

Recently, three cohort studies have investigated the relationship between dietary Mg intake and dementia risk in general populations, and the results have been inconsistent. While two of the studies (Japan,^(8)^ UK^(9)^) concluded that a lower intake of dietary Mg is associated with a higher risk of incident dementia, the remaining study (China^(10)^) reported contrasting findings, suggesting that a high dietary intake of Mg is associated with an elevated risk of dementia. There are also several methodological problems with study designs. The studies by Ozawa et al.^(8)^ and Luo et al.^(10)^ did not use validated dietary assessment methods for Mg intake, while that by Takeuchi et al.^(9)^ used a validated dietary assessment method (a 24-hour recall method) for Mg intake, but their energy-adjusted multivariate analysis showed an insignificant association between Mg intake and dementia risk. In addition, sample sizes of the studies by Ozawa et al. (*N* = 1081)^(8)^ and Luo et al. (*N* = 1565)^(10)^ were relatively small, which may be associated with Type I error. While the study by Takeuchi et al.^(9)^ had a large sample size (*N* = 161,376), it targeted as many as 23 nutrients, which may also be associated with Type I error. These methodological differences could result in inconsistencies in dietary intake data, thereby affecting the comparability and reliability of the findings. Therefore, there is a need for large-scale, high-quality cohort studies that provide a more comprehensive and accurate assessment of dietary intake, thus enhancing the reliability and validity of the results.

Since 2011, we have been conducting a large cohort study on age-related diseases, including dementia, comprising 14,364 participants aged 40–74 years at baseline.^(11)^ The present study aims to examine the association between dietary Mg intake and dementia risk among middle-aged and older Japanese individuals.

## Materials and methods

### Participants

**Figure 1.**
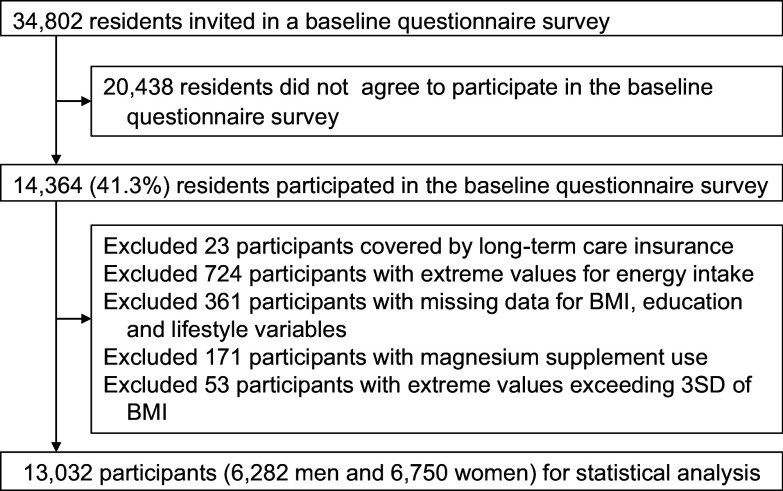
A flow chart of participant selection.

The Murakami cohort study is a population-based prospective cohort study of individuals aged 40–74 years living in Murakami city, Sekikawa village, and Awashimaura village (Niigata Prefecture, Japan).^(11)^ The Murakami cohort study targets an age range of 40–74 years, as it aims to ascertain risk factors for multiple outcomes of age- and lifestyle-related chronic diseases in middle-aged and older people. The present cohort study had an eight-year follow-up period. A flowchart of participant selection is shown in Figure 1. Of the total of 34,802 residents aged 40–74 years, 14,364 voluntarily participated in the baseline survey (2011–2013), during which questionnaires were distributed and collected through the community network. Of them, we excluded the following individuals: 23 participants who were covered by long-term care insurance (LTCI)^(12)^ at baseline (i.e., suspected of having mild cognitive decline or dementia), 724 who reported extreme values for energy intake (the Murakami cohort study excludes extreme values falling within the upper or lower 2.5% for dietary intake that may be unreliable^(13)^), 361 with missing data on education level, body mass index (BMI), energy intake, total physical activity (PA), smoking, drinking, or coffee consumption, 171 with Mg supplement use, and 53 outliers for BMI. The final number of analysed participants was 13,032 (90.7%). The protocol of the present study was approved by the Ethics Committee of Niigata University (Nos. 1324 and 2018-0417). Written informed consent was obtained from all participants.

### Baseline survey

The baseline survey was conducted using a self-administered questionnaire in 2011–2013. Data on sex, age, body weight, height, marital status, education level, occupation, lifestyle factors, supplement use, and disease history, including stroke, myocardial infarction, diabetes mellitus, and hypertension, were collected. Self-reported height and weight were validated against direct measurements in sub-samples of the study population. Spearman’s correlation coefficients between self-reported and measured values were high: 0.974 for male height, 0.975 for female height, 0.972 for male weight, and 0.973 for female weight (*N* = 1,752 males and 2,259 females; all *p* < 0.0001).^(11)^ Marital status was categorized as (1) married, (2) never married, and (3) divorced, separated, or bereaved; education level was categorized as (1) junior high school, (2) high school, (3) junior or vocational college, and (4) university; and occupation was categorized as (1) office work, sales and service work, (2) professional or management positions, (3) manual job (including security, farming, forestry, fishery, transportation, and labor services), (4) no job or housewife, and (5) others. Medication and supplement use, including product names, was self-reported. Participants were asked: ‘Is there any medication prescribed by your doctor that you take periodically?’ and ‘Are there any dietary health supplements that you have been taking with a frequency of once or more a week, continuously for one year or more?.’ Supplements containing Mg, including multivitamin/mineral supplements, were classified as Mg supplements. In addition, because laxatives generally contain Mg, they were also classified as Mg supplements. Disease history was based on self-reported physician diagnoses. Participants were asked, ‘Have you ever been diagnosed with any of the following illnesses by a doctor?’ and were provided with a list of specific conditions.

Participants were asked to complete a validated, 172-item self-administered food frequency questionnaire (FFQ)^(14)^ for estimating dietary intake. The FFQ used in this study was a semi-quantitative food frequency questionnaire, in which standard portion sizes were provided for each food item. The intake of each food was then estimated based on the reported frequency and portion size. Dietary Mg intake was calculated using the Standard Tables of Food Composition in Japan 2010^(15)^ by multiplying the nutrient content of each food by the frequency of its consumption and then summing the results of all food items. Spearman’s rank correlation coefficients between intakes estimated by the FFQ and those calculated using 12-day weighed food records for Mg were 0.39 for males and 0.51 for females.^(14)^ Total PA levels were assessed by calculating the metabolic equivalents (METs) score in MET-hours per day (MET-h/d). The questionnaire elicited the number of hours spent at each intensity level for sitting, standing, walking, and strenuous work for non-leisure-time PA; the frequency and duration at each intensity level for walking slowly, walking quickly, light to moderate exercise, and strenuous exercise for leisure-time PA; sleep; and other activities. To determine MET-h/d values, the daily duration of each activity was multiplied by its respective MET level. This method of measuring PA has been previously validated.^(16)^ Smoking habits were classified into four categories: (1) non-smoker, (2) past smoker, (3) 1 to 20 cigarettes per day, and (4) 20 or more cigarettes per day.^(17)^ Alcohol consumption was assessed by recording the frequency, usual amount, and type of alcohol consumed. Weekly ethanol consumption was calculated based on the daily amount and weekly frequency of ethanol intake. Alcohol consumption was then categorised into five groups: (1) non-drinker or rare drinker, (2) 1 to 149, (3) 150 to 299, (4) 300 to 449, and (5) 450 g or more ethanol per week.^(17)^ Coffee consumption (cups per day) was classified into five groups: (1) 0, (2) 0.1 to 0.9 (1–149 ml), (3) 1 to 1.9 (150–299 ml), (4) 2 to 2.9 (300–449 ml), and (5) 3 (≥450 ml) cups or more per day using a previously described method.^(18)^ BMI was calculated by dividing weight in kilograms (kg) by height in metres squared (m^2^). The protocol of the Murakami Cohort Study has been described in detail elsewhere.^(11)^

### Case finding

Incident dementia cases (up to March 31, 2020) were identified using the LTCI database over the eight-year follow-up period. The Japanese government introduced the LTCI system in 2000, and it has since become the nationwide framework for providing social care to frail and older individuals, both at home and in institutional settings.^(19)^ According to the LTCI dementia scale (Supplementary Table 1), disabling dementia is classified into six grades, ranging from 0 (no dementia) to V (severe dementia-related behavioral disturbance and cognitive impairment requiring medical treatment), based on the Doctor’s Opinion Paper of the physician in charge.^(20)^ Cases of incident dementia corresponded to grade II (moderate dementia-related behavioral disturbance and cognitive impairment with slight dependence) or higher.^(21)^ The accuracy of this dementia determination method has been reported to be high (94–97%).^(20)^ Detailed descriptions of this case-finding method have been provided previously.^(21)^ Person-years of observation were calculated using data on relocation and death from residency registration and death registration records in accordance with the Basic Residential Registry Law and the Family Registry Law.

### Statistical analysis

Baseline characteristics of participants were reported, with continuous variables presented as medians and interquartile ranges and categorical variables as numbers and percentages. Nutrient intake was adjusted for energy intake using the residual method.^(22)^ Participants were categorised into quartiles based on their energy-adjusted Mg intake. To determine the incidence rate of dementia, the number of dementia cases was divided by the total number of person-years. The Cox proportional hazard model was used to calculate both unadjusted and adjusted hazard ratios (HRs) for dementia across quartiles of energy-adjusted Mg intake. Covariates in the multivariable model included sex, age, marital status (dummy variable), education level, occupation (dummy variable), BMI,^(23)^ total PA level,^(24)^ smoking,^(17)^ alcohol consumption (coded as 1 for 0 or ≥450 g ethanol/week and 0 for other categories, as dementia risk is reportedly higher in the 0 and ≥450 groups^(17)^), coffee consumption,^(18)^ log-transformed energy intake, and histories of stroke, myocardial infarction, diabetes mellitus, and hypertension (dummy variables). For the subgroup analysis, HRs for dementia were calculated stratified by the absence or presence of disease history. Several sensitivity analyses were conducted regarding the association of Mg intake with dementia risk, including 1) the use of another potential dietary predictor, e.g., energy-adjusted total vegetable intake (Spearman’s correlation coefficient between this variable and energy-adjusted Mg intake quartile was 0.49) as an additional covariate, 2) excluding participants with a history of stroke, 3) excluding participants diagnosed with dementia before age 65 years, and those aged <58 years (a population not at risk of dementia in the present 8-year follow-up study), and 4) excluding cases of dementia that occurred within the first four years of follow-up to assess the possibility of reverse causation (i.e., individuals with early dementia may tend to have lower Mg intake). The cumulative incidence of dementia stratified by quartiles of Mg intake was determined by the Kaplan-Meier method. Statistical analysis was performed using SAS version 9.4 software (SAS Institute Inc., Cary, NC, USA). *P* < 0.05 was considered statistically significant.

## Results

The final study population comprised 6,282 males (48.2%) and 6,750 females (51.8%). The mean age was 59.0 years (SD 9.3), and the mean follow-up period was 8.1 years (SD 1.3). During the follow-up period, 286 cases of dementia occurred: 148 cases (2.4%) in males and 138 cases (2.0%) in females. Cases of dementia identified during the follow-up period included five participants in their 40s, 20 in their 50s, 113 in their 60s, and 148 in their 70s. Characteristics of participants at baseline according to quartiles of Mg intake by sex are presented in Table 1. Older age, higher total PA, higher coffee consumption, and a history of diabetes were significantly associated with higher quartiles of energy-adjusted Mg intake in both sexes. Conversely, having a manual job, current smoking, and drinking were inversely associated with higher quartiles of Mg intake in both sexes. Additionally, marital status, education level, and history of hypertension were correlated with higher Mg intake in females, and a history of stroke was specifically associated with higher Mg intake in males. Baseline participant characteristics for main food group consumption according to quartiles of energy-adjusted Mg intake are shown in Supplementary Table 2.

**Table 1.** Baseline participant characteristics according to quartile of energy-adjusted magnesium (Mg) intake

	Quartiles of energy-adjusted Mg intake				*P* for trend
Males					
	Q1 (*N* = 1571)	Q2 (*N* = 1570)	Q3 (*N* = 1571)	Q4 (*N* = 1570)	
Age (years)	58 (50–64)	60 (52–66)	61 (53–68)	62 (55–68)	<0.0001
Body mass index (kg/m^2^)	23.2 (21.3–25.5)	23.5 (21.6–25.6)	23.4 (21.5–25.2)	23.5 (21.8–25.4)	0.5522
Total energy (kcal/d)	2162 (1679–2643)	2252 (1825–2706)	2233 (1837–2734)	2111 (1723–2605)	0.6843
Total PA (MET-h/d)	44.8 (37.1–56.9)	43.3 (37.2–54.2)	43.3 (37.0–54.2)	43.3 (36.8–54.7)	0.0053
Mg intake (mg/day)	215 (162–274)	284 (227–345)	329 (267–404)	392 (317–498)	<0.0001
Married (%)	1227 (78.1)	1304 (83.1)	1308 (83.3)	1320 (84.1)	<0.0001
University graduates (%)	112 (7.1)	136 (8.7)	156 (9.9)	167 (10.6)	0.0003
Manual job (%)	518 (33.0)	481 (30.6)	478 (30.4)	435 (27.7)	0.0021
Current smoker (%)	660 (42.0)	541 (34.5)	462 (29.4)	439 (28.0)	<0.0001
Current drinker (%)	1384 (88.1)	1301 (82.9)	1263 (80.4)	1157 (73.7)	<0.0001
Coffee consumption^a^ (%)	598 (38.1)	787 (50.1)	909 (57.9)	1043 (66.4)	<0.0001
History of stroke (%)	28 (1.8)	40 (2.6)	38 (2.4)	65 (4.1)	0.0002
History of MI (%)	12 (0.8)	18 (1.2)	21 (1.3)	21 (1.3)	0.1130
History of diabetes (%)	130 (8.3)	145 (9.2)	163 (10.4)	226 (14.4)	<0.0001
History of hypertension (%)	375 (23.9)	372 (23.7)	387 (24.6)	364 (23.2)	0.8165
Females					
	Q1 (*N* = 1688)	Q2 (*N* = 1687)	Q3 (*N* = 1687)	Q4 (*N* = 1688)	
Age (years)	56 (47–64)	60 (52–67)	61 (54–67)	61 (55–67)	<0.0001
Body mass index (kg/m^2^)	22.5 (20.5–24.7)	22.3 (20.5–24.5)	22.5 (20.6–24.4)	22.3 (20.4–24.6)	0.1631
Total energy (kcal/d)	1806 (1429–2318)	1889 (1567–2343)	1906 (1552–2362)	1858 (1499–2287)	0.1834
Total PA (MET-h/d)	41.2 (36.8–51.0)	42.3 (37.3–50.7)	42.3 (37.2–50.8)	42.5 (37.8–51.4)	0.0601
Mg intake (mg/day)	234 (182–309)	296 (244–375)	340 (273–424)	404 (324–506)	<0.0001
Married (%)	1327 (78.6)	1383 (82.0)	1379 (81.7)	1355 (80.3)	0.2704
University graduates (%)	51 (3.0)	52 (3.1)	45 (2.7)	38 (2.3)	0.1267
Manual job (%)	282 (16.7)	255 (15.1)	242 (14.3)	226 (13.4)	0.0057
Current smoker (%)	161 (9.5)	102 (6.1)	86 (5.1)	115 (6.8)	0.0010
Current drinker (%)	640 (37.9)	558 (33.1)	580 (34.4)	566 (33.5)	0.0223
Coffee consumption^a^ (%)	653 (38.7)	845 (50.1)	1034 (61.3)	1219 (72.2)	<0.0001
History of stroke (%)	27 (1.6)	29 (1.7)	28 (1.7)	17 (1.0)	0.1655
History of MI (%)	2 (0.1)	1 (0.1)	6 (0.4)	0 (0)	0.8815
History of diabetes (%)	76 (4.5)	72 (4.3)	86 (5.1)	108 (6.4)	0.0065
History of hypertension (%)	268 (15.9)	315 (18.7)	349 (20.7)	305 (18.1)	0.0414

Data are shown as median with interquartile range in parentheses or number with percent in parentheses.

PA, physical activity; MET, metabolic equivalent; MI, myocardial infarction.

^a^Coffee consumption ≥ 150–299 ml/day.

Incidence rates and HRs for dementia according to quartiles of energy-adjusted Mg intake are shown in Table 2. For males, lower quartiles of energy-adjusted Mg intake were associated with a higher risk of dementia (multivariate-adjusted P for trend = 0.0410), with the lowest quartile having a significantly higher HR (1.73, 95% CI: 1.07–2.83) compared with the reference group (Q4). No associations were observed between quartiles of energy-adjusted Mg intake and the risk of dementia in females or the combined analysis of both sexes. The cumulative incidence of dementia as determined by the Kaplan-Meier method stratified by quartiles of energy-adjusted Mg intake is shown in Supplementary Figure 1.

**Table 2.** Incidence rates and hazard ratios (HRs) for dementia according to quartiles of energy-adjusted Mg intake by sex

	Quartiles of energy-adjusted Mg intake				*P* for trend
	Q1	Q2	Q3	Q4	
Males					
Number of participants	1571	1570	1571	1570	
Number of dementia cases	43	33	38	34	
Person-years (P-Y)	12,460	12,497	12,476	12,549	
Incidence rate (/1000P-Y)	3.5	2.6	3.0	2.7	
Unadjusted HR (95% CI)	1.27 (0.81–2.00)	0.97 (0.60–1.57)	1.13 (0.71–1.79)	1 (ref)	0.4060
Age-adjusted HR (95% CI)	1.88 (1.20–2.96)	1.23 (0.76–1.99)	1.21 (0.76–1.92)	1 (ref)	0.0085
Multivariate-adjusted HR^a^ (95% CI)	1.73 (1.07–2.83)	1.15 (0.70–1.88)	1.20 (0.75–1.93)	1 (ref)	0.0410
Females					
Number of participants	1688	1687	1687	1688	
Number of dementia cases	28	31	45	34	
Person-years (P-Y)	13,676	13,750	13,763	13,794	
Incidence rate (/1000P-Y)	2.0	2.3	3.3	2.5	
Unadjusted HR (95% CI)	0.83 (0.50–1.37)	0.91 (0.56–1.49)	1.33 (0.85–2.07)	1 (ref)	0.2389
Age-adjusted HR (95% CI)	1.18 (0.71–1.94)	1.01 (0.62–1.64)	1.35 (0.87–2.11)	1 (ref)	0.8289
Multivariate-adjusted HR^a^ (95% CI)	1.07 (0.63–1.82)	1.05 (0.63–1.73)	1.41 (0.89–2.23)	1 (ref)	0.9031
Total					
Number of participants	3259	3257	3258	3258	
Number of dementia cases	71	64	83	68	
Person-years (P-Y)	26,137	26,246	26,239	26,343	
Incidence rate (/1000P-Y)	2.7	2.4	3.2	2.6	
Unadjusted HR (95% CI)	1.05 (0.76–1.47)	0.94 (0.67–1.33)	1.23 (0.89–1.69)	1 (ref)	0.8322
Age-adjusted HR (95% CI)	1.53 (1.10–2.13)	1.12 (0.79–1.57)	1.28 (0.93–1.76)	1 (ref)	0.0377
Multivariate-adjusted HR^a^ (95% CI)	1.37 (0.96–1.96)	1.06 (0.75–1.51)	1.28 (0.93–1.76)	1 (ref)	0.1968

^a^Adjusted for age, sex, body mass index, marital status, education level, occupation, total physical activity levels, smoking, alcohol consumption, coffee consumption, total energy intake, and disease history (myocardial infarction, stroke, diabetes mellitus, and hypertension).

Multivariate-adjusted HRs for dementia according to quartiles of energy-adjusted Mg intake, including energy-adjusted total vegetable intake as an additional covariate, are presented in Supplementary Table 3 (sensitivity analysis). For males, lower quartiles of energy-adjusted Mg intake were associated with a higher risk of dementia (multivariate-adjusted *P* for trend = 0.0504), with the lowest quartile having a significantly higher multivariate-adjusted HR (1.82, 95% CI: 1.06–3.12) compared with the reference group.

Incidence rates and HRs for dementia according to quartiles of energy-adjusted Mg intake in males stratified by the absence or presence of disease history are shown in Table 3. Quartiles of energy-adjusted Mg intake were not significantly associated with the risk of dementia either in the group without a history of disease (multivariate-adjusted HR = 1.40, 95% CI: 0.73–2.69, P for trend = 0.2569) or in the group with a history of disease (multivariate-adjusted HR = 1.52, 95% CI: 0.74–3.11, *P* for trend = 0.3158). The *P*-value for the interaction between quartiles of energy-adjusted Mg intake and disease history on dementia risk was 0.9905. Incidence rates and HRs for dementia according to quartiles of energy-adjusted Mg intake in males, excluding those with a history of stroke, are presented in Table 4 (sensitivity analysis). Quartiles of energy-adjusted Mg intake were not significantly associated with the risk of dementia (multivariate-adjusted HR = 1.46, 95% CI: 0.89–2.42, P for trend = 0.1425).

**Table 3.** Incidence rates and hazard ratios (HRs) for dementia according to quartiles of energy-adjusted Mg intake in males stratified by the absence or presence of disease history^a^

	Quartiles of energy-adjusted Mg intake				*P* for trend
	Q1	Q2	Q3	Q4	
Absence of disease history^a^					
Number of participants	1060	1059	1058	1060	
Number of dementia cases	22	19	18	19	
Person-years (P-Y)	8510	8482	8479	8491	
Incidence rate (/1000P-Y)	2.6	2.2	2.1	2.2	
Unadjusted HR (95% CI)	1.15 (0.62–2.13)	1.00 (0.53–1.88)	0.95 (0.50–1.81)	1 (ref)	0.6267
Age-adjusted HR (95% CI)	1.77 (0.96–3.28)	1.30 (0.69–2.46)	1.03 (0.54–1.96)	1 (ref)	0.0534
Multivariate-adjusted HR^b^ (95% CI)	1.40 (0.73–2.69)	1.10 (0.57–2.12)	0.91 (0.47–1.75)	1 (ref)	0.2569
Presence of disease history^a^					
Number of participants	512	510	511	512	
Number of dementia cases	20	16	18	16	
Person-years (P-Y)	3947	4019	3997	4056	
Incidence rate (/1000P-Y)	5.1	4.0	4.5	3.9	
Unadjusted HR (95% CI)	1.29 (0.67–2.49)	1.01 (0.50–2.01)	1.14 (0.58–2.34)	1 (ref)	0.5393
Age-adjusted HR (95% CI)	1.80 (0.93–3.49)	1.18 (0.59–2.36)	1.28 (0.65–2.52)	1 (ref)	0.1161
Multivariate-adjusted HR^b^ (95% CI)	1.52 (0.74–3.11)	1.01 (0.50–2.08)	1.09 (0.55–2.17)	1 (ref)	0.3158

^a^History of myocardial infarction, stroke, diabetes mellitus, and hypertension.

^b^Adjusted for age, sex, body mass index, marital status, education level, occupation, total physical activity levels, smoking, alcohol consumption, coffee consumption, and energy intake.

**Table 4.** Incidence rates and hazard ratios (HRs) for dementia according to quartiles of energy-adjusted Mg intake in males, excluding those with a history of stroke

	Quartiles of energy-adjusted Mg intake				*P* for trend
	Q1	Q2	Q3	Q4	
Males					
Number of participants	1543	1530	1533	1505	
Number of dementia cases	39	28	31	32	
Person-years (P-Y)	12,265	12,205	12,192	12,040	
Incidence rate (/1000P-Y)	3.2	2.3	2.5	2.7	
Unadjusted HR (95% CI)	1.20 (0.75–1.91)	0.86 (0.52–1.43)	0.96 (0.58–1.57)	1 (ref)	0.5290
Age-adjusted HR (95% CI)	1.73 (1.09–2.77)	1.09 (0.66–1.82)	1.01 (0.62–1.66)	1 (ref)	0.0234
Multivariate-adjusted HR^a^ (95% CI)	1.46 (0.89–2.42)	0.95 (0.57–1.61)	0.93 (0.56–1.53)	1 (ref)	0.1425

^a^Adjusted for age, sex, body mass index, marital status, education level, occupation, total physical activity levels, smoking, alcohol consumption, coffee consumption, total energy intake, and disease history (myocardial infarction, diabetes mellitus, and hypertension).

Incidence rates and HRs for late-life dementia according to quartiles of energy-adjusted Mg intake among participants aged ≥58 years, excluding dementia cases diagnosed before age 65 years, are shown in Supplementary Table 4 (sensitivity analysis). In males, lower quartiles of energy-adjusted Mg intake were associated with a higher risk of dementia (multivariate-adjusted P for trend = 0.0495), with the lowest quartile having a significantly higher HR (1.80, 95% CI: 1.08–2.99) compared with the reference group.

When male participants who developed dementia within the first four years of follow-up were excluded from the analysis, lower quartiles of energy-adjusted Mg intake were marginally associated with a higher risk of dementia (multivariate-adjusted P for trend = 0.0596), with the lowest quartile demonstrating a significantly higher multivariate-adjusted HR (1.74, 95% CI: 1.01–3.00) compared with the reference group (Supplementary Table 5).

Mg intake according to main food group is shown in Supplementary Table 6. In males, the leading source of Mg intake was cereals (24.0%), followed by alcohol and non-alcohol beverages (19.2%), vegetables (13.6%), and beans (10.8%). In females, the leading source of Mg intake was vegetables (18.1%), followed by cereals (17.2%), alcohol and non-alcohol beverages (15.7%), and beans (11.3%).

## Discussion

The present study showed that lower dietary Mg intake is associated with an increased risk of dementia in middle-aged and older Japanese males, but not in females. Our results are consistent with some previous cohort studies, indicating that a low dietary Mg intake is associated with high dementia risk. Ozawa et al.^(8)^ suggested that the highest quartile of dietary Mg intake was potentially associated with a lower risk of all-cause dementia (HR 0.63, 95% CI: 0.40–1.01) than the lowest quartile in Japanese individuals. A study by Takeuchi et al.^(9)^ also showed similar associations across the UK Biobank population, finding that dietary Mg intake was associated with all-cause dementia (*P* for trend = 0.025), although the association was not statistically significant (*P* for trend = 0.111) when energy intake was taken into account. On the other hand, the results of other studies are not consistent with ours. The Shanghai Aging Study by Luo et al.^(10)^ reported a contradictory finding, indicating that higher dietary Mg intake is associated with an increased risk of incident dementia compared to the lowest intake used as a reference (HR 2.26, 95% CI: 1.02–5.00). This finding is difficult to understand but may be explained by the observation that overall Mg intake was low, even within the highest tertile group (>267.5 mg/day). Unfortunately, that study did not examine the observed effect stratified by sex. Lo et al.^(25)^ investigated the association between dietary Mg intake and the risk of mild cognitive impairment and/or probable dementia in postmenopausal women in the United States and found no association. Therefore, the association between dietary Mg intake and dementia remains unclear.

In the subgroup analysis by disease history (Table 3) and the sensitivity analysis (Table 4), the HR in the lowest Mg intake group in males was attenuated and was no longer statistically significant. This suggests that the observed association between Mg intake and dementia risk in males may be confounded by chronic diseases (in particular, stroke), given the higher incidence rates among those with such conditions. Although not statistically significant, the pattern of association between Mg intake and dementia risk in males was similar across both subgroups. Further studies will be needed to confirm this finding.

In the present study, an inverse association between Mg intake and dementia risk was found only in males. A study by Cherbuin et al.^(26)^ also reported a similar association only in males using mild cognitive impairment as an outcome. Although the reason for the observed sex difference is not entirely clear, it may be explained by the overall lower Mg intake in males relative to females in the present study (median intake of 300 mg/day for males and 317 mg/day for females). This difference should be larger if the sex difference in body size is taken into account. Moreover, the lack of an association between Mg intake and dementia risk in females may be explained by metabolism. The menopause transition and menopause are associated with increased bone resorption,^(27)^ during which Mg stored in bones may be released into the extracellular fluid^(28)^ more than in the premenopausal period. This age-related physiological phenomenon may decrease the risk of Mg deficiency in females. It has also been reported that there is no association between Mg intake and other chronic diseases, such as cancer and coronary heart disease in females.^(29,30)^

There are several possible mechanisms to explain how Mg insufficiency may contribute to the development of dementia. One potential pathway involves the regulation of NMDA receptors, which are essential for excitatory synaptic transmission, neuronal plasticity/density, and memory.^(31)^ Mg acts as a natural inhibitor of NMDA receptors, preventing overactivation that can lead to excitotoxicity, synaptic loss, and neuronal damage.^(32,33)^ Low Mg levels are associated with increased oxidative stress, promoting the generation of reactive oxygen species^(33)^ that damage cell membranes and mitochondrial function, further contributing to neurodegeneration.^(31)^ Mg also plays a critical role in modulating inflammatory responses, which contribute to tissue damage in neurodegenerative conditions driven by neuroinflammation.^(32)^ Low Mg levels are associated with increased production of pro-inflammatory cytokines such as IL-1, IL-6, and TNF-α, which are implicated in neuroinflammation and the progression of AD.^(31,33)^ Alateeq et al. reported that higher dietary Mg intake was significantly associated with lower inflammation levels across different inflammatory markers.^(34)^

In the present study population, the leading food groups contributing to Mg intake were cereals, beverages, vegetables, and beans, although the order differed between males and females. This finding is generally consistent with results of the Japanese nutrition survey using the dietary record method (Supplementary Table 7).^(35)^ Among these food groups, males had lower vegetable consumption than females, suggesting that increasing vegetable intake among males may help improve their Mg intake.

We estimated Mg intake based on food consumption; intake from drinking tap water was not assessed. However, it is also necessary to assess Mg intake from tap water as well as coffee and green tea, which are commonly consumed by middle-aged and older Japanese adults. Tap water in Japan is generally soft, and the tap water in Murakami City is no exception. The Mg concentration of tap water in Murakami city was determined to be as low as 2.4–6.7 mg/L in 2024 (data from the Murakami City Water and Sewage Division, unpublished). The average Mg concentration in tap water across Japan’s 47 prefectures has been reported to range from 1.0 to 7.0 mg/L (*SD*: 0.2–5.0),^(36)^ suggesting that the Mg concentration in the Murakami area is approximately in line with the national average.

The strength of the present cohort study lies in the utilisation of an appropriate method for assessing dietary Mg intake. Specifically, we employed an appropriate energy-adjusted method (residual method) with a validated FFQ to classify Mg intake (exposure levels). In addition, the sample size was large with a high follow-up rate.

The present study, however, has some limitations worth noting. First, data on dietary intake, sociodemographic characteristics, lifestyle factors, and disease history were obtained from a self-administered questionnaire, which may have introduced misclassification bias. Second, blood Mg levels were not available, and individual differences in absorption could have affected circulating Mg concentrations. This may have led to bias in the observed association between dietary Mg intake and dementia risk. Third, data on Mg supplementation during the follow-up period were not available. Fourth, incident dementia cases were identified using the LTCI database. Since some of these cases might not have been diagnosed by neurologists, misclassification of dementia cases could have attenuated the association between Mg intake and the risk of dementia. Although the specificity of the method used to diagnose dementia is reportedly high, ranging from 94% to 97%, its sensitivity is limited to 73%.^(20)^ This may have resulted in selection bias in identifying dementia cases. Fifth, dementia subtypes were not classified. Given that AD accounts for approximately two-thirds of dementia cases,^(37)^ our findings may primarily relate to AD. Sixth, while an association between dietary Mg intake and dementia risk was observed, this relationship might be influenced by reverse causation. For instance, early-stage dementia might have impacted eating behaviour and food preferences.^(38,39)^ Longer-term follow-up studies are essential to elucidate the causality of these associations. Finally, our results were not adjusted for all potential confounders, including genetic factors (e.g., APOE polymorphisms) and other dietary factors, and their changes over the follow-up period. Given these limitations, the present findings should be interpreted with caution. Furthermore, because the association between Mg intake and dementia risk was weak, limited to males, and appeared to be disease-dependent, further well-designed cohort studies will be needed to confirm this association.

In conclusion, the present cohort study revealed that low Mg intake is associated with a high risk of dementia in middle-aged and older Japanese males, but not in females. However, this association may be partly confounded by a history of disease. Considering that dietary risk factors are modifiable, lifestyle interventions aimed at improving Mg intake may help reduce the risk of dementia.

## Supporting information

Bulycheva et al. supplementary material 1 Bulycheva et al. supplementary material

Bulycheva et al. supplementary material 2 Bulycheva et al. supplementary material

## Supplementary material

The supplementary material for this article can be found at https://doi.org/10.1017/jns.2025.10075.
