# Supplementary figures and images for: Dietary magnesium intake and dementia risk in community-dwelling people aged 40–74 years: an 8-year cohort study
Source: J Nutr Sci. 2026 Jan 22;15:e12. doi: 10.1017/jns.2025.10075 (PMC12854930; doi:10.1017/jns.2025.10075)

## Slide 1
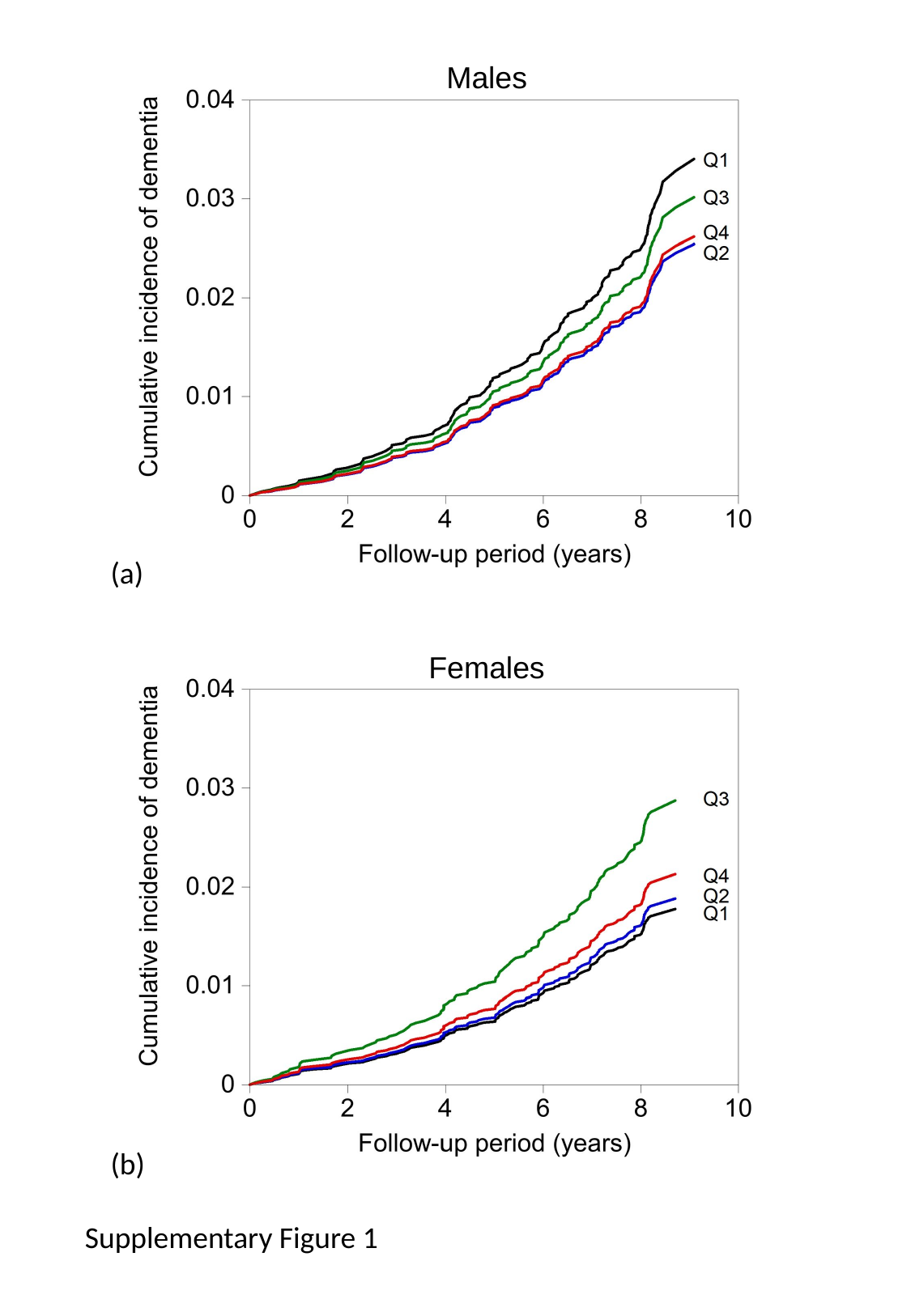

Males
(a)
Females
(b)
Supplementary Figure 1

Supplement: Bulycheva et al. supplementary material 1 — Bulycheva et al. supplementary material [file S204867902510075Xsup001.pptx]
