# Supplementary material for: Dietary magnesium intake and dementia risk in community-dwelling people aged 40–74 years: an 8-year cohort study
Source: J Nutr Sci. 2026 Jan 22;15:e12. doi: 10.1017/jns.2025.10075 (PMC12854930; doi:10.1017/jns.2025.10075)
Supplement: Bulycheva et al. supplementary material 2 — Bulycheva et al. supplementary material [file S204867902510075Xsup002.docx]

**Supplementary Files**

Supplementary Table 1. Dementia scale used in the long-term care insurance (LTCI) system

Grade 0: No dementia, fully independent.

Grade I: The individual has some level of dementia but is largely independent in daily life, both at home and in social settings.

Grade II: Although the individual may exhibit some symptoms, behaviors, or communication difficulties that interfere with daily life, they can live independently as long as someone provides occasional supervision or attention.

Grade III: The individual intermittently exhibits symptoms, behaviors, or communication difficulties that interfere with daily life and requires caregiving support.

Grade IV: The individual frequently exhibits symptoms, behaviors, or communication difficulties that interfere with daily life and requires constant caregiving.

Grade V: The individual presents with severe psychiatric symptoms, problematic behaviors, or serious physical illness, and requires specialized medical care.

Notification from the Director-General of the Health and Welfare Bureau for the Elderly, Ministry of Health and Welfare, October 26, 1993 (https://www.mhlw.go.jp/topics/kaigo/kentou/15kourei/sankou4.html)

| Supplementary Table 2. Consumption of main food groups according to quartiles of energy-adjusted magnesium (Mg) intake | | | | | |
| --- | --- | --- | --- | --- | --- |
|  | **Quartiles of energy-adjusted Mg intake** | | | | P for trend |
| **Males** | | | | | |
|  | Q1 (N=1571) | Q2 (N=1570) | Q3 (N=1571) | Q4 (N=1570) |  |
| Cereals (g/day) | 600 (510-766) | 626 (541-759) | 604 (510-728) | 571 (447-677) | <.0001 |
| Potatoes (g/day) | 12 (3-25) | 22 (11-37) | 29 (15-49) | 29 (15-58) | <.0001 |
| Beans (g/day) | 23 (10-41) | 38 (23-64) | 52 (30-82) | 62 (32-106) | <.0001 |
| Vegetables (g/day) | 94 (45-156) | 158 (100-241) | 228 (133-334) | 252 (141-407) | <.0001 |
| Fruits (g/day) | 42 (10-95) | 93 (45-164) | 125 (59-221) | 141 (60-260) | <.0001 |
| Mushrooms (g/day) | 3 (0-6) | 6 (3-13) | 9 (4-15) | 8 (4-16) | <.0001 |
| Seaweed (g/day) | 2 (1-5) | 4 (2-8) | 6 (3-11) | 6 (3-13) | <.0001 |
| Fish and shellfish (g/day) | 44 (17-77) | 61 (34-101) | 67 (30-84) | 62 (34-107) | <.0001 |
| Meats (g/day) | 49 (22-95) | 53 (31-93) | 52 (30-84) | 41 (21-68) | <.0001 |
| Eggs (g/day) | 25 (11-39) | 25 (11-50) | 25 (11-39) | 25 (11-39) | 0.0147 |
| Milk and dairy products (g/day) | 35 (5-121) | 99 (20-210) | 130 (36-246) | 130 (26-246) | <.0001 |
| **Females** | | | | | |
|  | Q1 (N=1688) | Q2 (N=1687) | Q3 (N=1687) | Q4 (N=1688) | <.0001 |
| Cereals (g/day) | 502 (424-598) | 500 (424-581) | 484 (409-550) | 439 (346-516) | <.0001 |
| Potatoes (g/day) | 25 (14-42) | 37 (23-59) | 41 (25-67) | 43 (24-71) | <.0001 |
| Beans (g/day) | 31 (17-53) | 47 (29-76) | 61 (34-98) | 74 (40-125) | <.0001 |
| Vegetables (g/day) | 168 (106-248) | 255 (176-363) | 297 (204-430) | 371 (233-577) | <.0001 |
| Fruits (g/day) | 117 (53-212) | 180 (95-302) | 204 (115-340) | 213 (111-364) | <.0001 |
| Mushrooms (g/day) | 6 (3-13) | 11 (6-19) | 13 (6-19) | 13 (6-24) | <.0001 |
| Seaweed (g/day) | 3 (1-6) | 6 (3-9) | 6 (3-11) | 7 (4-15) | <.0001 |
| Fish and shellfish (g/day) | 49 (27-80) | 66 (42-103) | 69 (42-110) | 65 (38-103) | <.0001 |
| Meats (g/day) | 53 (29-90) | 48 (28-79) | 44 (24-70) | 35 (18-58) | <.0001 |
| Eggs (g/day) | 25 (11-39) | 25 (11-39) | 25 (11-39) | 25 (11-39) | 0.0012 |
| Milk and dairy products (g/day) | 121 (43-230) | 166 (75-292) | 183 (71-320) | 150 (56-274) | 0.0002 |

| Supplementary Table 3. Incidence rates and hazard ratios (HRs) for dementia according to quartiles of energy-adjusted Mg intake by sex, additionally adjusted for vegetable intake | | | | | |
| --- | --- | --- | --- | --- | --- |
|  | Quartiles of energy-adjusted Mg intake | | | | P for trend |
|  | Q1 | Q2 | Q3 | Q4 |  |
| **Males** | | | | | |
| Multivariate-adjusted HR* (95% CI) | 1.82 (1.06-3.12) | 1.18 (0.71-1.95) | 1.21 (0.76-1.94) | 1 (ref) | 0.0504 |
| **Females** | | | | | |
| Multivariate-adjusted HR* (95% CI) | 0.82 (0.46-1.47) | 0.93 (0.56-1.55) | 1.31 (0.82-2.07) | 1 (ref) | 0.3143 |
| **Total** | | | | | |
| Multivariate-adjusted HR* (95% CI) | 1.23 (0.83-1.81) | 1.01 (0.71-1.44) | 1.25 (0.90-1.74) | 1 (ref) | 0.5610 |

*Adjusted for age, sex, body mass index, marital status, education level, occupation, total physical activity levels, smoking, alcohol consumption, coffee consumption, energy intake, disease history (myocardial infarction, stroke, diabetes, and hypertension), and energy-adjusted vegetable intake.

| Supplementary Table 4. Incidence rates and hazard ratios (HRs) for late-life dementia according to quartiles of energy-adjusted Mg intake among participants aged ≥58 years, excluding cases diagnosed before age 65 years | | | | | |
| --- | --- | --- | --- | --- | --- |
|  | Quartiles of energy-adjusted Mg intake | | | | P for trend |
|  | Q1 | Q2 | Q3 | Q4 |  |
| **Males** | | | | | |
| Number of participants | 786 | 892 | 997 | 1068 |  |
| Number of dementia cases | 38 | 28 | 36 | 32 |  |
| Person-years (P-Y) | 6053 | 6949 | 7810 | 8469 |  |
| Incidence rate (/1000P-Y) | 6.3 | 4.0 | 4.6 | 3.8 |  |
| Unadjusted HR (95% CI) | 1.68 (1.05-2.68) | 1.07 (0.64-1.77) | 1.23 (0.76-1.97) | 1 (ref) | 0.0625 |
| Age-adjusted HR (95% CI) | 1.87 (1.69-3.00) | 1.15 (0.69-1.91) | 1.22 (0.76-1.97) | 1 (ref) | 0.0175 |
| Multivariate-adjusted HR* (95% CI) | 1.80 (1.08-2.99) | 1.09 (0.65-1.84) | 1.24 (0.76-2.01) | 1 (ref) | 0.0495 |
| **Females** | | | | | |
| Number of participants | 785 | 1010 | 1055 | 1099 |  |
| Number of dementia cases | 24 | 31 | 40 | 32 |  |
| Person-years (P-Y) | 6289 | 8187 | 8565 | 8936 |  |
| Incidence rate (/1000P-Y) | 3.8 | 3.8 | 4.7 | 3.6 |  |
| Unadjusted HR (95% CI) | 1.07 (0.63-1.81) | 1.06 (0.65-1.73) | 1.31 (0.82-2.08) | 1 (ref) | 0.9968 |
| Age-adjusted HR (95% CI) | 1.09 (0.64-1.86) | 1.08 (0.66-1.77) | 1.28 (0.80-2.03) | 1 (ref) | 0.8899 |
| Multivariate-adjusted HR* (95% CI) | 1.00 (0.57-1.76) | 1.12 (0.67-1.86) | 1.30 (0.81-2.11) | 1 (ref) | 0.9093 |
| **Total** | | | | | |
| Number of participants | 1571 | 1902 | 2051 | 2167 |  |
| Number of dementia cases | 62 | 59 | 76 | 64 |  |
| Person-years (P-Y) | 12342 | 15136 | 16375 | 17405 |  |
| Incidence rate (/1000P-Y) | 5.0 | 3.9 | 4.6 | 3.7 |  |
| Unadjusted HR (95% CI) | 1.38 (0.97-1.95) | 1.06 (0.75-1.51) | 1.26 (0.91-1.76) | 1 (ref) | 0.1753 |
| Age-adjusted HR (95% CI) | 1.48 (1.04-2.09) | 1.11 (0.78-1.58) | 1.25 (0.90-1.74) | 1 (ref) | 0.0669 |
| Multivariate-adjusted HR* (95% CI) | 1.35 (0.93-1.96) | 1.08 (0.75-1.55) | 1.26 (0.90-1.77) | 1 (ref) | 0.2249 |

*Adjusted for age, sex, body mass index, marital status, education level, occupation, total physical activity levels, smoking, alcohol consumption, coffee consumption, energy intake, and disease history (myocardial infarction, stroke, diabetes mellitus, hypertension).

| Supplementary Table 5. Incidence rates and hazard ratios (HRs) for dementia according to quartiles of energy-adjusted Mg intake in males, after excluding dementia cases occurring within the first four years of follow-up | | | | | |
| --- | --- | --- | --- | --- | --- |
|  | Quartiles of energy-adjusted Mg intake | | | | P for trend |
|  | Q1 | Q2 | Q3 | Q4 |  |
| **Males** | | | | | |
| Number of participants | 1565 | 1559 | 1558 | 1563 |  |
| Number of dementia cases | 37 | 22 | 25 | 27 |  |
| Person-years (P-Y) | 12449 | 12477 | 12440 | 12533 |  |
| Incidence rate (/1000P-Y) | 3.0 | 1.8 | 2.0 | 2.2 |  |
| Unadjusted HR (95% CI) | 1.38 (0.84-2.27) | 0.82 (0.46-1.43) | 0.93 (0.54-1.61) | 1 (ref) | 0.2444 |
| Age-adjusted HR (95% CI) | 2.01 (1.22-3.30) | 1.02 (0.58-1.80) | 1.00 (0.58-1.72) | 1 (ref) | 0.0080 |
| Multivariate-adjusted HR* (95% CI) | 1.74 (1.01-3.00) | 0.91 (0.51-1.63) | 0.97 (0.56-1.69) | 1 (ref) | 0.0596 |

*Adjusted for age, sex, body mass index, marital status, education level, occupation, total physical activity levels, smoking, alcohol consumption, coffee consumption, total energy intake, and disease history (myocardial infarction, stroke, diabetes mellitus, hypertension).

| Supplementary Table 6. Average intake of magnesium (Mg) according to main food group | | | |
| --- | --- | --- | --- |
| Food group | Mg intake (mg/day) | | |
|  | **Males (319)** | **Females (340)** | **Total (330)** |
| Cereals | 76.5 (24.0%) | 58.4 (17.2%) | 67.0 (20.3%) |
| Potatoes | 5.2 (1.6%) | 7.6 (2.2%) | 6.4 (1.9%) |
| Sugars and sweeteners | 0.0 (0.0%) | 0.0 (0.0%) | 0.0 (0.0%) |
| Beans | 34.5 (10.8%) | 38.6 (11.3%) | 36.6 (11.1%) |
| Nuts and seeds | 4.3 (1.3%) | 3.9 (1.2%) | 4.1 (1.2%) |
| Vegetables | 43.5 (13.6%) | 61.7 (18.1%) | 53.0 (16.1%) |
| Fruits | 15.1 (4.7%) | 25.1 (7.4%) | 20.3 (6.2%) |
| Mushrooms | 1.5 (0.5%) | 2.3 (0.7%) | 1.9 (0.6%) |
| Seaweed | 10.2 (3.2%) | 11.9 (3.5%) | 11.1 (3.4%) |
| Fish and shellfish | 26.4 (8.3%) | 26.3 (7.7%) | 26.3 (8.0%) |
| Meats | 12.1 (3.8%) | 10.9 (3.2%) | 11.5 (3.5%) |
| Eggs | 3.9 (1.2%) | 3.6 (1.1%) | 3.7 (1.1%) |
| Milk and dairy products | 18.1 (5.7%) | 26.2 (7.7%) | 22.4 (6.8%) |
| Confectionaries | 5.0 (1.6%) | 8.7 (2.5%) | 6.9 (2.1%) |
| Alcohol and non-alcohol beverages | 61.2 (19.2%) | 53.2 (15.7%) | 57.0 (17.3%) |
| Seasonings and spices | 1.4 (0.4%) | 1.5 (0.4%) | 1.4 (0.4%) |

| Supplementary Table 7. Average intake of magnesium (Mg) according to food group in individuals aged ≥20 years (National Health and Nutrition Survey 2023, Japan) | |
| --- | --- |
| Food group | Mg intake (mg/day) |
| Cereals | 42.8 (17.3%) |
| Potatoes | 7.1 (2.9%) |
| Sugars and sweeteners | 0.1 (0.0%) |
| Beans | 35.6 (14.4%) |
| Nuts and seeds | 5.7 (2.3%) |
| Vegetables | 32.2 (13.0%) |
| Fruits | 11.1 (4.5%) |
| Mushrooms | 1.4 (0.6%) |
| Seaweed | 6.6 (2.7%) |
| Fish and shellfish | 19.0 (7.7%) |
| Meats | 19.7 (8.0%) |
| Eggs | 4.5 (1.8%) |
| Milk and dairy products | 13.0 (5.3%) |
| Confectionaries | 4.9 (2.0%) |
| Alcohol beverages | 4.0 (1.6%) |
| Alcohol and non-alcohol beverages | 19.3 (7.8%) |
| Seasonings and spices | 24.0 (9.7%) |
